# Evaluation of pulpal anesthesia and injection pain using IANB with pre-heated, buffered and conventional 2% lignocaine in teeth with symptomatic irreversible pulpitis—a randomized clinical study

**DOI:** 10.7717/peerj.14187

**Published:** 2022-10-19

**Authors:** Namita Gandhi, Nimisha Shah, Dian Agustin Wahjuningrum, Sweetly Purnomo, Riana Nooshian, Suraj Arora, Ajinkya M. Pawar

**Affiliations:** 1 Department of Conservative Dentistry and Endodontics, K M Shah Dental College and Hospital, Sumandeep Vidyapeeth, Vadodara, Gujarat, India; 2 Department of Conservative Dentistry, Faculty of Dental Medicine, Universitas Airlingga, Surabaya City, East Java, Indonesia; 3 Department of Conservative Dentistry and Endodontics, Nair Hospital Dental College, Mumbai, Maharashtra, India; 4 Department of Restorative Dental Sciences, College of Dentistry, King Khalid University, Abha, Saudi Arabia

**Keywords:** Buffered, Local anesthesia, Pre-warm, Irreversible pulpitis, Endodontics

## Abstract

**Background:**

The efficacy of 2% lignocaine is reduced in a hot tooth. Local aesthetic agents can be preheated and buffered to increase their effectiveness. The present investigation was carried out due to limited information concerning adult patients with symptomatic irreversible pulpitis in mandibular teeth.

**Methods:**

A total of 252 individuals were included in the clinical trial in accordance with the selection criteria only after clinical study was registered with the Clinical Trial Registry of India (CTRI/2020/09/027796). Scores on the visual analog scale (VAS) and electric pulp test (EPT) on a 1–10 scale were recorded prior to the commencement of therapy. In this double-blinded study, patients were randomly divided by a co-investigator using computer randomisation (www.randomizer.org) into three groups, group A: inferior alveolar nerve blocks (IANB) with 2% lignocaine preheated at 42 °C (injected at 37 °C) (*N* = 84), group B: IANB of 2% lignocaine buffered with 0.18 ml of 8.4% sodium bicarbonate (*N* = 80) and group C: 2% lignocaine (*N* = 88). Excluding the dropouts of individuals (*n* = 11), wherein the anaesthesia failed, a total of 241 people were finally assessed 15 minutes after profound anaesthesia, endodontic access, and intraoperative pain were quantified using VAS. Pain on injection for all three groups was recorded immediately after IANB with VAS. The analysis was performed using one way ANOVA with Tukey’s post hoc test and Paired T-Test using SPSS version 21.

**Results:**

Preheated, Buffered, and conventional 2% lignocaine showed statistically significant reduction in intraoperative pain (*P* < 0.001) compared to pre-operative but on inter-group comparison preheated and buffered showed highly significant pain reduction compared with conventional 2% lignocaine (*P* < 0.001).

**Conclusions:**

Warm and buffered local anaesthetic (LA) were effective in reducing intraoperative discomfort than conventional LA. Preheated local anesthetics caused the least pain, followed by buffered local anesthetics, while conventional local anesthetics caused the most pain.

## Introduction

In order to minimise discomfort during different dental, endodontic, and minor surgical treatments, local anaesthetic (LA) is necessary (Queiroz et al., 2015). In the majority of patients, it is challenging to achieve enough anaesthetic success for a “hot” tooth. According to the literature, inferior alveolar nerve blocks (IANB) using lignocaine in mandibular posterior teeth had a failure rate of 44%–81% (Claffey et al., 2004; Potočnik & Bajrović, 1999). There are a number of causes, including local tissue acidosis brought on by the production of lactic acid and its by-products, hyperalgesia offered on by inflamed pulp, and a lower resting membrane potential, but the most widely accepted theory is that tetrodotoxin-resistant sodium channels are to penalise (TTXr). Lignocaine makes it four times harder for these channels to close, and inflammation doubles the production of these molecules (Wells et al., 2007; Badrian et al., 2016).

Changes in injection method (Meechan, 1999), supplemental anaesthesia techniques (Yadav, 2015; Bhalla, Taneja & Chockattu, 2021), changes in anaesthetic liquid, etc. (Nagendrababu et al., 2019) are a few of the approaches utilised to increase the success rate of IANB in hot teeth. Lignocaine containing adrenaline usually have a pH range between 2.9–4.4 (Malamed, Tavana & Falkel, 2013). This pH is recommended to prolong the shelf life and to prevent oxidation of LA, but at the same time it shows reduction in its efficacy, burning sensation, slow anesthesia onset.

When used for mandibular or maxillary anaesthesia, elevating the pH of lignocaine by neutralising it with 8.4% sodium bicarbonate accelerates the dissociation rate and increases the concentration of uncharged base ions crossing the nerve membrane (Kattan et al., 2019).

Warming LA to 42 °C is another effective way to boost its effectiveness (Aravena et al., 2018; Tirupathi & Rajasekhar, 2020; Hogan et al., 2011). The LA molecule may infiltrate the nociceptor, causing sodium channels to block more promptly. This could be the result of local anaesthetics’ temperature-dependent, decreasing pKa (dissociation constant) value (Allen, Bunce & Presland, 2008). According to Powell (1987), lignocaine has a pKa of 7.57 at 40 °C and 7.92 at 25 °C. As a result, warming lignocaine may expedite the initiation of local anaesthetic and enhance its effectiveness.

The speed, location, and pH of the anaesthetic solution are only a few of the many aspects of local anaesthesia delivery that might induce pain. As a result, patients get anxious and postpone away necessary surgeries. A research by Gümüş & Aydinbelge (2019) demonstrated that pre-warming LA decreases injection discomfort. In a similar context, Palanivel et al. (2020) revealed that buffered LA caused the least discomfort during administration.

Since there is sporadic literature comparing the efficacy of preheated, buffered, and conventional LA on adult population, the present double-blinded randomized clinical study was designed aiming to evaluate the pulpal anesthesia and injection pain using IANB with pre-heated, buffered and conventional 2% lignocaine in teeth with symptomatic irreversible pulpitis. The null hypothesis was that there is no difference in efficacy of pulpal anesthesia and injection pain using IANB with pre-heated, buffered and conventional 2% lignocaine in teeth with symptomatic irreversible pulpitis.

## Materials and Method

### Study design, ethical approval, and clinical trial registry

This double-blind randomized clinical study was approved by the Sumandeep Vidyapeeth Institutional Ethics Committee (SVIEC/ON/DentBNPG18/D19047; date of approval 22/11/2019), India. The protocol was developed and registered at the clinical trial registry of India (CTRI/2020/09/027796). The current superiority trial was reported according to Consolidated Standards of Reporting Trials (CONSORT) guidelines (Schulz, Altman & Moher, 2010). Written informed consent was obtained from all participants in this study.

### Sample size

In a one-way ANOVA study, sample sizes of minimum 60, 60 and 60 were obtained from the three groups whose means were compared. The total sample of 180 subjects achieves 80% power to detect differences among the means *versus* the alternative of equal means using an F test with a 0.05 significance level. The size of the variation in the means is represented by their standard deviation which is 30.0 the common standard deviation within a group is assumed to be 1.13. Between groups, the one way analysis of variance with multiple comparison tested at 5% level. The sample size formula used was: (Zalpha +Zbeta)^2^*Sqrt(n*delta^2^/2kS^2^), where Zalpha = 1.96; Zbeta = 0.84; n = total number of groups = 3; delta = mean difference = 30.0; k = degrees of freedom = n-1 = 2; S = standard deviation = 1.13.

However number of patients enrolled in the study were 252 divided into in following three groups: (A) preheated 2% lignocaine, (*n* = 84); (B) buffered local anesthesia, (*n* = 80); and (C) conventional 2% lignocaine, (*n* = 88).

### Selection criteria

Patients were selected as per the inclusion: patients among 18 to 60 years of age with mandibular hot teeth (Symptomatic irreversible pulpitis), having actively experienced moderate to severe pain on a visual analog scale (VAS) scale of five or more were included in the study. Exclusion criteria: Patients with known hypersensitivity to Lignocaine and sodium bicarbonates, who had undergone cardiac surgery in the last six months, pregnant or lactating females, or with necrosed teeth with sinus or swelling, severe periodontitis and poor oral hygiene, cracks, fracture, and open apex were excluded from the study.

### Randomization and allocation concealment

A postgraduate student assessed the eligibility of five hundred and twenty-one patients based on clinical examinations, radiographs, and pulp sensibility tests. Clinically tooth having spontaneous/lingering pain/nocturnal pain with moderate to deep carious lesion and absences of tenderness on percussion and delayed response to the electric pulp test (EPT) were taken for further radiographic examination. Tooth with radiolucency involving enamel, dentin, and approaching pulp was selected. All the radiographs were taken with a long cone and paralleling technique using a positioning indicator device. Two hundred and fifty-two patients meet the selection criteria and agreed to participate in the trial. Co-investigator implemented the random sequence generation and allocation concealment. Randomization was done by computer randomization (https://www.randomizer.org/) and patients were assigned into three groups.

The allocation concealment ratio was 1:1:1. This was done by inserting the LA cartridges in sequentially numbered sealed opaque envelopes. The envelopes were marked with the randomization code. As soon as the patient was placed in the intervention group, the number was noted in the patient’s case sheet and decoded at the end of the trial. 

### Blinding

The entire procedure was double-blinded to avoid bias. The primary investigator and the patient both were blinded to the groups allotted. The operator directly received an aspirating metal syringe loaded with the cartridge of lignocaine; pre-heated lignocaine or buffered lignocaine with a 27-gauge needle attached to the tip of the unit.

### Clinical procedure

Patients were sensitized to a (1 to 10-point) VAS scale. This scale was given to the patient to choose thrice: the first time was before the injection, second time after receiving the injection, and the third after entering the pulp chamber and a pre-operative VAS score was recorded. Pre-operative pulp sensibility test was recorded using the electric pulp test (EPT). The patient was explained about the test and the tooth was checked first followed by the affected tooth. Patients were asked to indicate when a tingling sensation occurs to him/her, and the response of the affected tooth was noted down in numbers.

For group A—The preparation of preheated local anesthesia was done according to method described by Allen, Bunce & Presland (2008) and Davidson & Boom (1992). A 1.8 ml cartridge of commercially accessible 2% lignocaine hydrochloride with 1:80,000 adrenaline (Lignospan special, Septodont Healthcare India) was placed in a composite warmer (12 VDC, 2000Mpa, 24W0 power supply; AR Heat), for 4 min. Two cartridges were placed in the heating slot of the warmer and the thermostat is set in such a way that a temperature of 42 °C was obtained for the anesthetic liquid. The rubber cap of the second cartridge was removed and a thermometer was used to check the temperature of the anesthetic solution, as it is ascertained at 37 °C (body temp), the first 1.8 ml cartridge was administered to the patient.

For group B—The preparation of buffered local anesthesia was done according to a previous study (Saatchi et al., 2015). The buffered local anesthetic solution has a shelf-life of one week, but it was prepared fresh once every two days for maximum efficacy. Under sterile conditions, 0.18 ml from a 1.8-ml cartridge of 2% Lignocaine with 1:80,000 adrenaline was drawn and replaced with 0.18 ml 8.4% sodium bicarbonate using a 1 ml plastic syringe and stored in the refrigerator. The cartridge was inverted five times to mix the solution. As a result, no precipitation was formed. It was shaken until the solution was clear; this ensured that the sodium bicarbonate was completely dissolved. The cartridge was then loaded into a metal syringe and injected.

For group C—Preparation of conventional group – Conventional nerve block with 1.8ml of 2% lignocaine with 1:80,000 adrenaline was injected. IANB in all the three experimental groups was given with a metal syringe with 27-G, a 1.5-inch needle attached to a standard aspirating dental injection syringe about 1 mm, and 1.8 ml of the solution was deposited slowly (2 min). Immediately after injection, VAS was used to evaluate the injection pain for all the experimental groups.

All the patients were asked to wait for 15 min for the profound anesthesia to be achieved. Subjective symptoms like tingling sensation, numbness of lower lip, buccal and lingual periosteum on the respective side of jaw were considered, whereas objective symptoms like EPT (Parkell Gentel Pulse vitality tester) of concerned tooth was done, negative response to EPT was considered as effective anesthesia. Those patients who do not showed subjective and objective symptoms were given supplementary intra-ligamentary injections and were excluded from the study (consort flow chart).

Isolation was performed with the help of a rubber dam fifteen minutes after the injection. Excavation of caries was done along the walls of the tooth and lastly, the pulpal roof was prepared. Access cavity preparation was done with help of endo access bur to design the access cavity. After entering the pulp chamber and intra-operative VAS score was recorded as intra-operative reading. Further, the endodontic treatment was performed as per the standard methods and protocol by the primary investigator.

### Statistical methods

The obtained data were tabulated and statistically analyzed using SPSS version 21 and *p*-value and Chi-square Value, one way ANOVA with Tukey’s post hoc test, and paired t-test were applied. For the statistical test between the group, a one-way analysis of variance with multiple comparison tests at the level of significance was set as 5%.

## Results

### Demographic data

**Figure 1:**
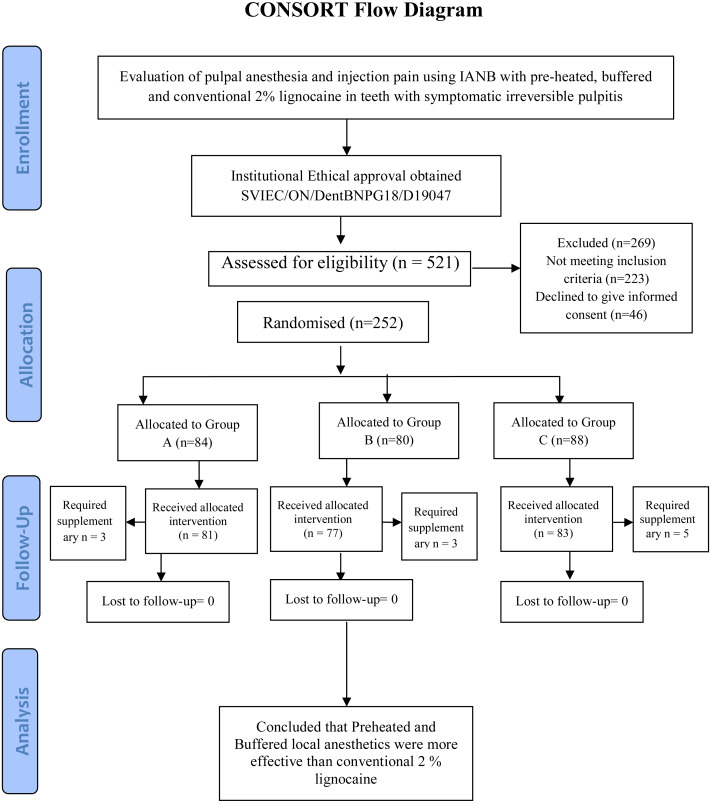
CONSORT 2010 flow diagram.

The patients enrolled in the clinical trial are presented on the CONSORT 2010 flow diagram (Fig. 1). Total of 252 patients were included in present study of which 11 patients were dropped out as lip numbness was not achieved after 15 min of INAB and considered as failure due to the wrong technique. So, 241 patients were included for final evaluation. Out of the total enrolled patients, 119 were male, while 122 were female. The age of 41 patients was between (18–25) years of age, 82 patients were between (26–36) years, 66 patients were between (37–46) years of age and the remaining 52 patients were between (47–60) years of age.

### Pre-Intra operative VAS score

The mean pre-operative pain using a 10-mm Visual Analog Scale (VAS) was 7.28 mm ± 1.26 mm, for Group A. For Group B mean VAS score was 6.88 mm ± 1.23 mm, and for Group C score was 6.88 mm ± 1.24 mm (Table 1). On comparing the means of all three groups no statistical difference was found in the pre-operative pain values. While the mean of Intra-operative pain for Group A was 1.59 mm ± 1.03 mm, for Group B 1.69 mm ± 1.07 mm, and Group C was 3.54 mm ± 2.34 mm. This shows that all three local anesthetic agents were highly effective in reducing pain (*P* value <0.001).

**Table 1:** Comparison of visual analog scale (VAS) pre and post with paired *t*-test for groups after excluding the drop-outs during the clinical trial.

Groups		*N* = 241	Mean	Std. deviation	Mean difference	*t*-value	*p*-value
Group A	VAS Pre	81	7.28	1.26	5.69	36.075	<0.001
	VAS Post	81	1.59	1.03			
Group B	VAS Pre	77	6.88	1.23	5.18	38.120	<0.001
	VAS Post	77	1.69	1.07			
Group C	VAS Pre	83	6.88	1.24	3.34	12.331	<0.001
	VAS Post	83	3.54	2.34			

Table 2 shows an inter-group comparison between all the three experimental groups for the reduction in intra-operative pain, there was no statistically significant difference (*P* = 0.183) between Group A (Preheated LA) and Group B (Buffered LA). Whereas there was a highly significant difference (*P* < 0.001) between Group A (Preheated LA)—Group C (Conventional LA) and between Group B (buffered LA)—Group C (Conventional LA). This indicates that buffered and preheated local anesthetic solutions are more efficient in reducing pain in patients with symptomatic irreversible pulpitis in comparison to conventional 2% local anesthetic agents.

**Table 2:** Comparison between the groups for vas score (pre-intra) by Tukey’s post hoc test.

**Dependent variable**	**Group**		**Mean difference**	**Std. Error**	***p*-value**
VAS Difference (pre-intra)	Group-A	Group-B	0.51	0.28	0.183
		Group-C	2.35	0.28	<0.001^*^
	Group-B	Group-C	1.84	0.28	<0.001^**^

**Notes.**

The values marked with (* and **) exhibited significant difference.

### Pain on injection

The mean pain on LA administration using VAS (Visual Analog Scale) for Group A was 1.35 mm ± 1.09 mm, Group B was 2.08 mm ± 1.27 mm, and Group C was 3.19 mm ± 0.93 mm. Table 3 shows the mean difference between Group A and Group B was −0.73 mm ± 0.17 mm and between Group A and Group C was −1.84 mm ± 0.17 mm stating that there statistically significant difference between the groups (*P* value <0.001). Correspondingly comparing Group B with Group C showed a mean difference of −1.11 mm ± 0.17 mm and a *p*-value of <0.001 thus indicating there was a statistically significant difference between them concerning pain on injection. This shows that preheated LA showed the least pain on injection followed by buffered and conventional LA.

**Table 3:** Comparison between the groups for pain on injection (VAS SCORE).

**Dependent variable**	**Group**		**Mean difference**	**Std. Error**	***p*-value**
Pain on injection	Group-A	Group-B	−0.73	0.17	<0.001
		Group-C	−1.84	0.17	<0.001
	Group-B	Group-C	−1.11	0.17	<0.001

**Notes.**

*P* values 0.05 are corellated with significant difference.

## Discussion

In the current clinical exploration, patients with symptomatic irreversible pulpitis were evaluated to determine the effectiveness of inferior alveolar nerve block in relieving pain using pre-heated, buffered, and standard 2% lignocaine. The study’s null hypothesis was rejected in light of the findings.

Clinical dentistry has changed from being an unpleasant and traumatic experience to one that is substantially less uncomfortable and more satisfying because to the efficacious use of LA. Profound anaesthesia during root canal therapy not only helps the patient but also frees the dentist from worrying about unanticipated movements or reactions from the patient. Patients with symptomatic irreversible pulpitis (hot tooth) and challenges with mandibular teeth sometimes have trouble achieving enough anaesthetic effect (Sahu, Kabra & Choudhary, 2019). Therefore, amendments are suggested to increase efficacy.

Changing the pH and temperature of the anaesthetic solution is the most productive technique to improve efficacy and lessen pain during injection, according to a clinical trial on minors (Kurien, Goswami & Singh, 2018). Warming the local anaesthetic solution to body temperature (37 °C) before administration seemed to lessen pain during intraoral local anaesthesia administration (Aravena et al., 2018; Tirupathi & Rajasekhar, 2020) and buffered local anaesthetic (Kattan et al., 2019) solutions in adult patients, according to a number of randomised clinical studies and systematic reviews on prewarmed and unwarmed LA solution. However, there is scant information comparing preheated, buf. So the current study was created.

The Visual Analog Score was used to assess the decrease in intra-operative pain and pre-operative discomfort. Because the VAS is dependable, repeatable, and simpler for patients to comprehend and record, we chose to utilise it (Hawker et al., 2011).

The effectiveness of IANB is often assessed by the subjective and objective symptoms that patients experience after being under anaesthesia, however an electric pulp tester (EPT) is a more accurate way to assess pulpal anaesthesia (Warren et al., 2017). Progressive pulpal anaesthesia is defined as no response to EPT. Contrasted with the study by Certosimo & Archer (1996), which demonstrated that a “no reaction” at an 80-reading guaranteed pulpal anaesthesia in crucial asymptomatic teeth For a longer shelf life, anaesthetic solutions sold commercially are acidic (Malamed, Tavana & Falkel, 2013). Unfortunately, the LA solution’s acidity has several drawbacks that affect how well it works in clinical settings, so we need to modify it. Buffering local anaesthesia is one such improvement. It is made by mixing 1.8 ml of LA with 0.18 ml of sodium bicarbonate, 8.4%, which results in the creation of carbon dioxide and water (Afsal et al., 2019). Since carbon dioxide directly depresses the axon, concentrates LA into the nerve trunk (ion trapping), and changes LA into an active cationic state, it helps buffered LA work more effectively.

In patients with a hot tooth, buffering LA enhances the chance of effective anaesthesia by 2.29 times, according to a systematic review by Kattan et al. (2019). Kurien, Goswami & Singh (2018) and Saatchi et al. (2018) both endorse the same. However, Schellenberg et al. (2015) and Hobeich et al. (2013) reported dissenting findings. Different populations involved, non-standard buffering approaches, varying injection methodologies, and various assessment techniques can all lead to variances (Palanivel et al., 2020).

Pre-heating local anaesthetic at 42 °C is another method for increasing LA effectiveness in inflamed pulp (Afsal et al., 2019). By blocking sodium channels, conventional LA prevents a change in the nerve impulse’s course of propagation. By increasing membrane fluidity, which makes it easier for lignocaine to pass and reach the effective concentration faster, and by densely expressing TRPV1 channels in trigeminal tissue, warming at 42 °C aids in faster blockage of the sodium channels (Afsal et al., 2019). According to Alonso, Perula & Rioja (1993), there was a negative correlation between temperature and pain, with 10 °C having the greatest mean pain level and the following temperatures: 18 °C, 37 °C, and 42 °C. In order to prevent any negative reactions from happening to the oral tissue, pre-heated LA was administered at 37 °C, or at the physiological tissue pH. According to Davidson & Boom (1992), subcutaneous infusion of LA at body temperature (37 °C) lowers pain severity after minor oral surgery.

In this investigation, the warmed group’s intra-operative agony was much lower than it was in the traditional LA group. There were no significant differences between pre-warmed and traditional LA, which was in contrast to Ram, Hermida & Peretz (2002) but in conformity with Tirupathi & Rajasekhar (2020) and Aravena et al. (2018). The modified Behavioral Pain Scale (BPS), which is difficult to comprehend, was employed as the evaluation criterion, which may have contributed to the disparity between the research populations. The secondary result of pain during injection was investigated, and preheated and buffered 2% lignocaine was shown to cause the least discomfort. This finding was consistent with a clinical investigation by Gümüş & Aydinbelge (2019).

The study’s shortcoming is that just one concentration of sodium bicarbonate (8.4%) was utilised to buffer LA; more research carried out using different concentrations is warranted. The same research design must be used to analyse patients with systemic disorders (such as hypertension, diabetes mellitus, and other systemic illnesses).

## Conclusions

Considering the limitations of the study, we would like to conclude that preheated, buffered, and conventional local anesthesia was effective in reducing pain in symptomatic irreversible pulpitis. When compared to standard LA, the warmed and buffered LA was more successful in reducing intraoperative discomfort. Preheated local anaesthetics and buffered local anaesthetics caused the least amount of discomfort during administration, but the standard group caused higher pain. Future RCTs with a larger sample size will be beneficial to confirm the findings.

##  Supplemental Information

10.7717/peerj.14187/supp-1 Supplemental Information 1 Master-chart raw data

## Additional Information and Declarations

### Data Availability

The following information was supplied regarding data availability: The raw measurements are available as a Supplemental File.

### Clinical Trial Registration

The following information was supplied regarding Clinical Trial registration: CTRI/2020/09/027796.

## References

[ref-1] Afsal MM, Khatri A, Kalra N, Tyagi R, Khandelwal D (2019). Pain perception and efficacy of local analgesia using 2% lignocaine, buffered lignocaine, and 4% articaine in pediatric dental procedures. Journal of Dental Anesthesia and Pain Medicine.

[ref-2] Allen MJ, Bunce C, Presland AH (2008). The effect of warming local anaesthetic on the pain of injection during sub-tenon’s anaesthesia for cataract surgery. Anaesthesia.

[ref-3] Alonso PE, Perula LA, Rioja LF (1993). Pain-temperature relation in the application of local anaesthesia. British Journal of Plastic Surgery.

[ref-4] Aravena PC, Barrientos C, Troncoso C, Coronado C, Sotelo-Hitschfeld P (2018). Effect of warming anesthetic on pain perception during dental injection: a split-mouth randomized clinical trial. Local and Regional Anesthesia Volume.

[ref-5] Badrian H, Modaresi J, Davoudi A, Sabzian R (2016). Irreversible pulpitis and achieving profound anesthesia: complexities and managements. Anesthesia: Essays and Researches.

[ref-6] Bhalla VK, Taneja S, Chockattu SJ (2021). Failure of molar anesthesia in endodontics: a systematic review. Saudi Endodontic Journal.

[ref-7] Certosimo AJ, Archer RD (1996). A clinical evaluation of the electric pulp tester as an indicator of local anesthesia. Operative Dentistry.

[ref-8] Claffey E, Reader A, Nusstein J, Beck M, Weaver J (2004). Anesthetic efficacy of articaine for inferior alveolar nerve blocks in patients with irreversible pulpitis. Journal of Endodontics.

[ref-9] Davidson JA, Boom SJ (1992). Warming lignocaine to reduce pain associated with injection. BMJ.

[ref-10] Gümüş H, Aydinbelge M (2019). Evaluation of effect of warm local anesthetics on pain perception during dental injections in children: a split-mouth randomized clinical trial. Clinical Oral Investigations.

[ref-11] Hawker GA, Mian S, Kendzerska T, French M (2011). Measures of adult pain: visual Analog Scale for pain (Vas Pain), numeric rating scale for pain (NRS Pain), McGill Pain Questionnaire (MPQ), short-form mcgill pain questionnaire (SF-MPQ), chronic pain grade scale (CPGS), short form-36 bodily pain scale (SF.). Arthritis Care & Research.

[ref-12] Hobeich P, Simon S, Schneiderman E, He J (2013). A prospective, randomized, double-blind comparison of the injection pain and anesthetic onset of 2% lidocaine with 1:100, 000 epinephrine buffered with 5% and 10% sodium bicarbonate in maxillary infiltrations. Journal of Endodontics.

[ref-13] Hogan M-E, vanderVaart S, Perampaladas K, Machado M, Einarson TR, Taddio A (2011). Systematic review and meta-analysis of the effect of warming local anesthetics on injection pain. Annals of Emergency Medicine.

[ref-14] Kattan S, Lee S-M, Hersh EV, Karabucak B (2019). DO buffered local anesthetics provide more successful anesthesia than nonbuffered solutions in patients with pulpally involved teeth requiring dental therapy?. The Journal of the American Dental Association.

[ref-15] Kurien RS, Goswami M, Singh S (2018). Comparative evaluation of anesthetic efficacy of warm, buffered and conventional 2% lignocaine for the success of inferior alveolar nerve block (IANB) in mandibular primary molars: a randomized controlled clinical trial. Journal of Dental Research, Dental Clinics, Dental Prospects.

[ref-16] Malamed SF, Tavana S, Falkel M (2013). Faster onset and more comfortable injection with alkalinized 2% lignocaine with adrenaline 1:100,000. Compendium of Continuing Education in Dentistry.

[ref-17] Meechan JG (1999). How to overcome failed local anaesthesia. British Dental Journal.

[ref-18] Nagendrababu V, Duncan HF, Whitworth J, Nekoofar MH, Pulikkotil SJ, Veettil SK, Dummer PM (2019). Is articaine more effective than lidocaine in patients with irreversible pulpitis? an umbrella review. International Endodontic Journal.

[ref-19] Palanivel I, Ramakrishnan K, Narayanan V, Gurram P (2020). A prospective, randomized, double-blinded, cross over comparison of buffered versus non-buffered 2% lidocaine with 1:80, 000 adrenaline for dental extraction. International Journal of Applied Dental Sciences.

[ref-20] Potočnik I, Bajrović F (1999). Failure of inferior alveolar nerve block in endodontics. Dental Traumatology.

[ref-21] Powell MF (1987). Pharmaceutical Research.

[ref-22] Queiroz AM, Carvalho AB, Censi LL, Cardoso CL, Leite-Panissi CR, Silva RA, Carvalho FK, Nelson-Filho P, Silva LA (2015). Stress and anxiety in children after the use of computerized dental anesthesia. Brazilian Dental Journal.

[ref-23] Ram D, Hermida LB, Peretz B (2002). A comparison of warmed and room-temperature anesthetic for local anesthesia in children. Pediatric Dentistry.

[ref-24] Saatchi M, Khademi A, Baghaei B, Noormohammadi H (2015). Effect of sodium bicarbonate–buffered lidocaine on the success of inferior alveolar nerve block for teeth with symptomatic irreversible pulpitis: a prospective, randomized double-blind study. Journal of Endodontics.

[ref-25] Sahu S, Kabra P, Choudhary E (2019). Hot tooth—a challenge to endodontists. International Journal of Science and Research.

[ref-26] Saatchi M, Shafiee M, Khademi A, Memarzadeh B (2018). Anesthetic efficacy of gow-gates nerve block, inferior alveolar nerve block, and their combination in mandibular molars with symptomatic irreversible pulpitis: a prospective, randomized clinical trial. Journal of Endodontics.

[ref-27] Schellenberg J, Drum M, Reader A, Nusstein J, Fowler S, Beck M (2015). Effect of buffered 4% lidocaine on the success of the inferior alveolar nerve block in patients with symptomatic irreversible pulpitis: a prospective, randomized, double-blind study. Journal of Endodontics.

[ref-28] Schulz KF, Altman DG, Moher D (2010). Consort 2010 statement: updated guidelines for reporting parallel group randomised trials. Journal of Pharmacology and Pharmacotherapeutics.

[ref-29] Tirupathi SP, Rajasekhar S (2020). Effect of warming local anesthesia solutions before Intraoral Administration in dentistry: a systematic review. Journal of Dental Anesthesia and Pain Medicine.

[ref-30] Warren VT, Fisher AG, Rivera EM, Saha PT, Turner B, Reside G, Phillips C, White RP (2017). Buffered 1% lidocaine with epinephrine is as effective as non-buffered 2% lidocaine with epinephrine for mandibular nerve block. Journal of Oral and Maxillofacial Surgery.

[ref-31] Wells JE, Bingham V, Rowland KC, Hatton J (2007). Expression of nav1.9 channels in human dental pulp and trigeminal ganglion. Journal of Endodontics.

[ref-32] Yadav S (2015). Anesthetic success of supplemental infiltration in mandibular molars with irreversible pulpitis: a systematic review. Journal of Conservative Dentistry.

